# Melt inclusion vapour bubbles: the hidden reservoir for major and volatile elements

**DOI:** 10.1038/s41598-020-65226-3

**Published:** 2020-06-03

**Authors:** Swetha Venugopal, Federica Schiavi, Severine Moune, Nathalie Bolfan-Casanova, Timothy Druitt, Glyn Williams-Jones

**Affiliations:** 1 Department of Earth Sciences, Simon Fraser University, Burnaby, Canada; 2 Laboratoire Magmas et Volcans, Université Clermont Auvergne, CNRS, IRD, OPGC, Clermont-Ferrand, France; 3 Observatoire Volcanologique et Sismologique de Guadeloupe (OVSG), Gourbeyre, FWI Guadeloupe; 4 Institut de Physique du Globe de Paris, Université de Paris, Paris, France

**Keywords:** Geochemistry, Mineralogy, Petrology, Volcanology

## Abstract

Olivine-hosted melt inclusions (MIs) provide samples of magmatic liquids and their dissolved volatiles from deep within the plumbing system. Inevitable post-entrapment modifications can lead to significant compositional changes in the glass and/or any contained bubbles. Re-heating is a common technique to reverse MI crystallisation; however, its effect on volatile contents has been assumed to be minor. We test this assumption using crystallised and glassy basaltic MIs, combined with Raman spectroscopy and 3D imaging, to investigate the changes in fluid and solid phases in the bubbles before and after re-heating. Before re-heating, the bubble contains CO_2_ gas and anhydrite (CaSO_4_) crystallites. The rapid diffusion of major and volatile elements from the melt during re-heating creates new phases within the bubble: SO_2_, gypsum, Fe-sulphides. Vapour bubbles hosted in naturally glassy MIs similarly contain a plethora of solid phases (carbonates, sulphates, and sulphides) that account for up to 84% of the total MI sulphur, 80% of CO_2_, and 14% of FeO. In both re-heated and naturally glassy MIs, bubbles sequester major and volatile elements that are components of the total magmatic budget and represent a “loss” from the glass. Analyses of the glass alone significantly underestimates the original magma composition and storage parameters.

## Introduction

Olivine-hosted melt inclusions (MIs) provide insight into the nature of the magma mantle source, storage conditions, and pre-eruptive volatile contents^1^. Following entrapment, MIs undergo compositional modifications due to growth of the host olivine along the MI walls, and to crystallisation of daughter minerals from the glass due to slow ascent rates, and/or cooling^1,2^. Another modification is the nucleation of a vapour bubble in response to decompression during cooling and post-entrapment crystallisation^1,2^, further reducing the solubility of volatiles in the glass. Vapour, or shrinkage, bubbles produced by differential thermal contraction between the melt (glass) and the host crystal are considered to be inherent to the MI^1,2^. However, pre-existing bubbles that formed externally in a vapour-saturated system may also become trapped inside MIs. Vapour bubbles may also form during MI leakage and decrepitation of the host crystal^3,4^. Discriminating between various bubble types depends upon the size of the bubble relative to the total inclusion. Since the volumetric proportions of vapour bubbles depend on the cooling rate, volatile content and melt composition, cooling-related shrinkage and melt-saturated bubbles normally comprise 0.2 to 10 vol% of the inclusion^3,4^. Bubbles with greater volumetric proportions are not considered inherent to the MI^3,4^.

Due to the strong pressure-dependency of CO_2_ solubility, the contraction of the melt and the decrease of the internal pressure in response to cooling and post-entrapment crystallisation first leads to rapid CO_2_ saturation of the melt, and consequently, the transfer of CO_2_ gas into the bubble^5–7^, so that analyses of the glass yield erroneously low magmatic CO_2_ concentrations. This poses significant problems as the CO_2_ content is commonly used to infer the pressure of crystallisation and MI entrapment, as well as magmatic storage depths. By only considering the glass, these values are grossly underestimated.

Many of the post-entrapment modifications that occur within olivine-hosted MIs can be corrected using well-constrained exchange coefficients and recently established methods to quantify the amount of volatiles, particularly CO_2_, sequestered by the bubble. These approaches include the use of trace element proxies, such as CO_2_/Nb, to determine the pre-eruptive CO_2_ content of the undegassed melt^8^, reheating the MI to resorb the bubble ﻿and retrieve the CO_2_ content of the MI at the time of entrapment^9–11^, and estimating the mass of CO_2_ present in the bubble with Raman spectroscopy and adding it back into the glass^4,7,8,10,12,13^. Initial estimates concluded that 40 to 90% of the total CO_2_ occurs in the vapour bubble^4,7,10,13^. However, Raman spectroscopy has also revealed the presence of carbonates in some bubbles^14–16^, suggesting the percentage of original CO_2_ in the bubble may be much higher^4,15,17,18^. Other solid phases have also been observed within MI-hosted bubbles, including gypsum and native sulphur, presenting similar issues of sequestration for sulphur^16^. The presence of H_2_O in MI bubbles has also been reported in liquid form and in gypsum, nahcolite, and hydrous silicate solids^17^.

In the case of crystallised MIs, experimental methods include homogenisation and re-heating to dissolve the crystals and retrieve the initial magma composition. Although the effects of re-heating on the H_2_O content and *f*O_2_ of the MI have been previously examined^19,20^, the impact on major and other volatile elements has been assumed to be minor, and remains untested. Furthermore, the composition of vapour bubbles in crystallised MIs has only been studied following re-heating, with the major assumption that all phases in the bubble were present prior to re-heating^16^. We investigate this assumption using primitive (Fo > 80 mol%) basaltic olivine-hosted MIs from three volcanoes along the Garibaldi Volcanic Belt in western Canada (Mount Cayley, Garibaldi Lake and Mount Meager). We address two main questions. (1) Does re-heating modify the composition of MI bubbles? To track the compositional changes induced by re-heating, we analysed the vapour bubbles of eight naturally crystallised MIs from Mount Cayley and Garibaldi Lake by Raman spectroscopy before and after re-heating. (2) How does the vapour bubble contribute to the total volatile budget of the MI? This was addressed through Raman analyses of the bubble of seven glassy MIs from Mount Meager to examine the migration of C–O–H–S volatiles from the glass to the bubble during natural cooling. For both questions, selected bubbles were scanned in 3D using Raman spectroscopy.

Here we show that MI vapour bubbles are reservoirs for major and volatile elements that diffuse from the melt/glass. Over a range of magmatic and post-eruptive temperatures (1200 °C to surface conditions), S, H_2_O, CO_2_, Fe, Ca and Mg diffuse from the glass and form new phases within the bubble. It follows that analyses of only the glassy portions of MIs will significantly underestimate the magma composition and yield incorrect storage parameters (T, P, Fe^3+^/ΣFe, *f*O_2_).

### Sample selection

The Garibaldi Volcanic Belt (GVB) is a dormant volcanic arc that lies northward of the High Cascades along the western margin of North America. Volcanism along the GVB results from the slow (~45 mm yr^−1^) and oblique subduction of the Juan de Fuca Plate beneath the North American Plate^21^. The dominant eruptive composition along the arc is intermediate calc-alkaline, with basaltic eruptions comprising a small volume fraction^22^. Whole rock data are found in Supplementary Table S1. We chose primitive (Fo > 80 mol%) basaltic olivine-hosted MIs that have been thoroughly investigated for their major, volatile and trace element contents and magmatic conditions from Mount Cayley, Garibaldi Lake and Mount Meager^21^. These samples were chosen based on their arc-typical compositions, the lack of a brine phase during magmatic evolution^21^, which would otherwise affect the distribution and composition of phases in the vapour bubble^13^, and their well-constrained mantle source estimations^21^.

Glassy basaltic MIs hosted in unzoned olivine phenocrysts (Fo 77–84 mol%) are ubiquitous in the Pleistocene-aged breccia deposits from Mount Meager^21^. All MIs sampled from Mount Cayley lava flows (Fo 86-89 mol%; 12 -10 ka)^21^, and tephra deposits from The Cinder Cone (Fo 79-88 mol%; 40 ka)^22^ within the Garibaldi Lake Volcanic Field, are crystallised and hosted in unzoned olivines. Based on the emplacement mechanism of these deposits, MI crystallisation was likely a syn-eruptive process due to slow cooling.

Trace element modelling suggests the sub-arc mantle beneath Mount Cayley, Garibaldi Lake and Mount Meager is best represented by the depleted MORB mantle^23^ (DMM) modified by 2 to 10 wt% of hydrous fluids derived from the Juan de Fuca Plate, followed by 5 to 12% partial melting^21^. Based on these factors, the composition of the MIs, and subsequently their vapour bubble, can be considered representative of primitive arc magma. Finally, these MIs provide several technical benefits wherein the clarity of the vapour bubble, size of the MIs (<30 μm), and orientation of daughter minerals allowed for easier and more efficient Raman and 3D acquisitions.

**Figure 1:**
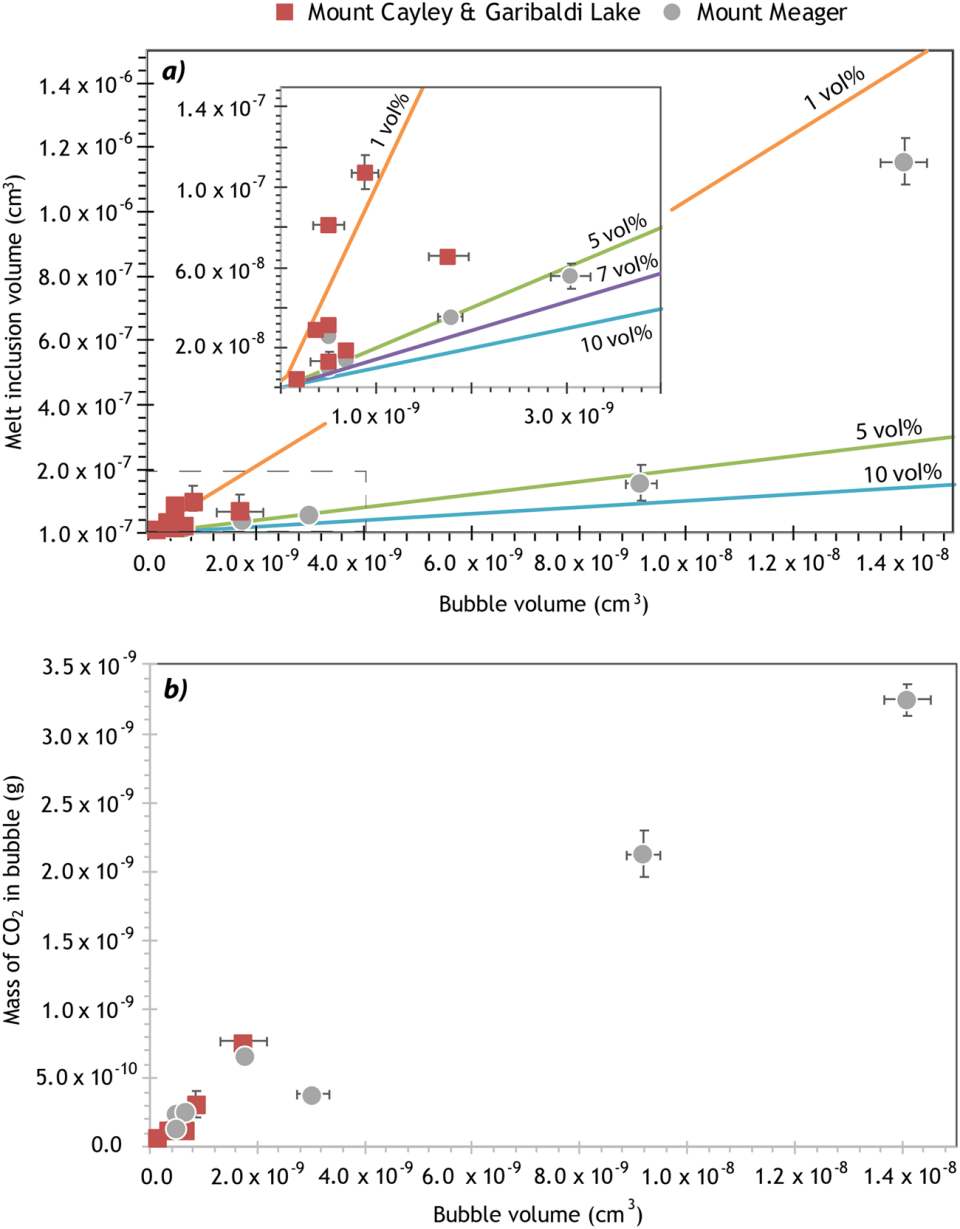
Comparing the volume of the vapour bubble to the corresponding (**a**) MI volume and (**b**) mass of CO_2_ in the bubble for all samples in this study. Inset in (**a**) refers to the area indicated by the grey dashed box. Coloured lines indicate volume ratio between the bubble and the MI; typically, the bubble comprises between 0.2 and 10 vol %. Volumetric proportions larger than this suggest the bubble is secondary, or pre-existing, and likely formed due to MI leakage, and/or rupturing of the host crystal. On both graphs, Mount Cayley and Garibaldi Lake data refer to values before re-heating. The mass of CO_2_ in the bubble was calculated using the Raman spectra, and assuming that CO_2_ is the only phase present in the bubble. Overall, the linear correlation suggests that every vapour bubble in this study is primary, was nucleated within the MI, and contains CO_2_ gas that was exsolved from the glass. One Mount Meager MI has a lower ratio of bubble to MI volume (MMA200; 1.2 vol%) due to a large MI volume, but a bubble size that is globally consistent. Error bars are 2σ and, in most samples, correspond to the size of the data marker.

All MIs in this study yield a positive relationship between MI and vapour bubble size (<10%), and a linear correlation between the vapour bubble size and the mass of CO_2_ in the bubble (Fig. 1). This suggests that the bubbles are inherent to the MI system, were nucleated at the time of decompression, and hence contain CO_2_ that was originally dissolved in the melt. One glassy MI has a lower bubble:MI volume ratio (MMA200; 1.2 vol%) due to a large MI volume, but a bubble size that is globally consistent.

### Re-heated MIs

**Figure 2:**
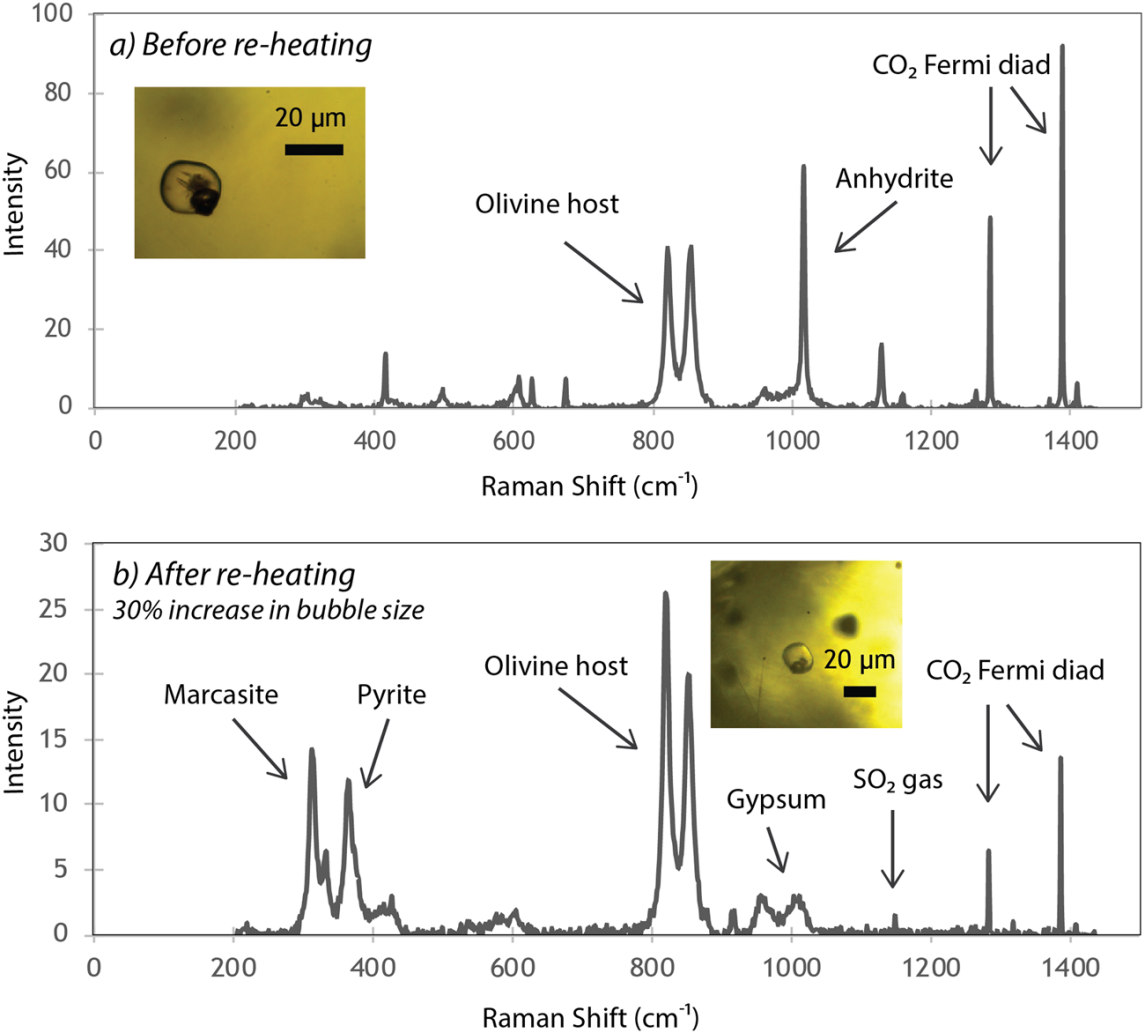
Raman spectra of vapour bubbles (**a**) before and (**b**) after re-heating. (**a**) Raman spectrum before re-heating of the bubble shown in the inset (Brhm-33). The two phases present are CO_2_ gas and anhydrite solids. (**b**) Raman spectrum of the same bubble after re-heating. The volume of the bubble increases, as well as the mass of CO_2_ within the bubble (see Table 1 and Supplementary Table S2). However, the total density of CO_2_ decreases. Anhydrite present before re-heating hydrates to gypsum. Additional S enters the bubble from the glass and forms pyrite, marcasite and SO_2_ gas.

**Figure 3:**
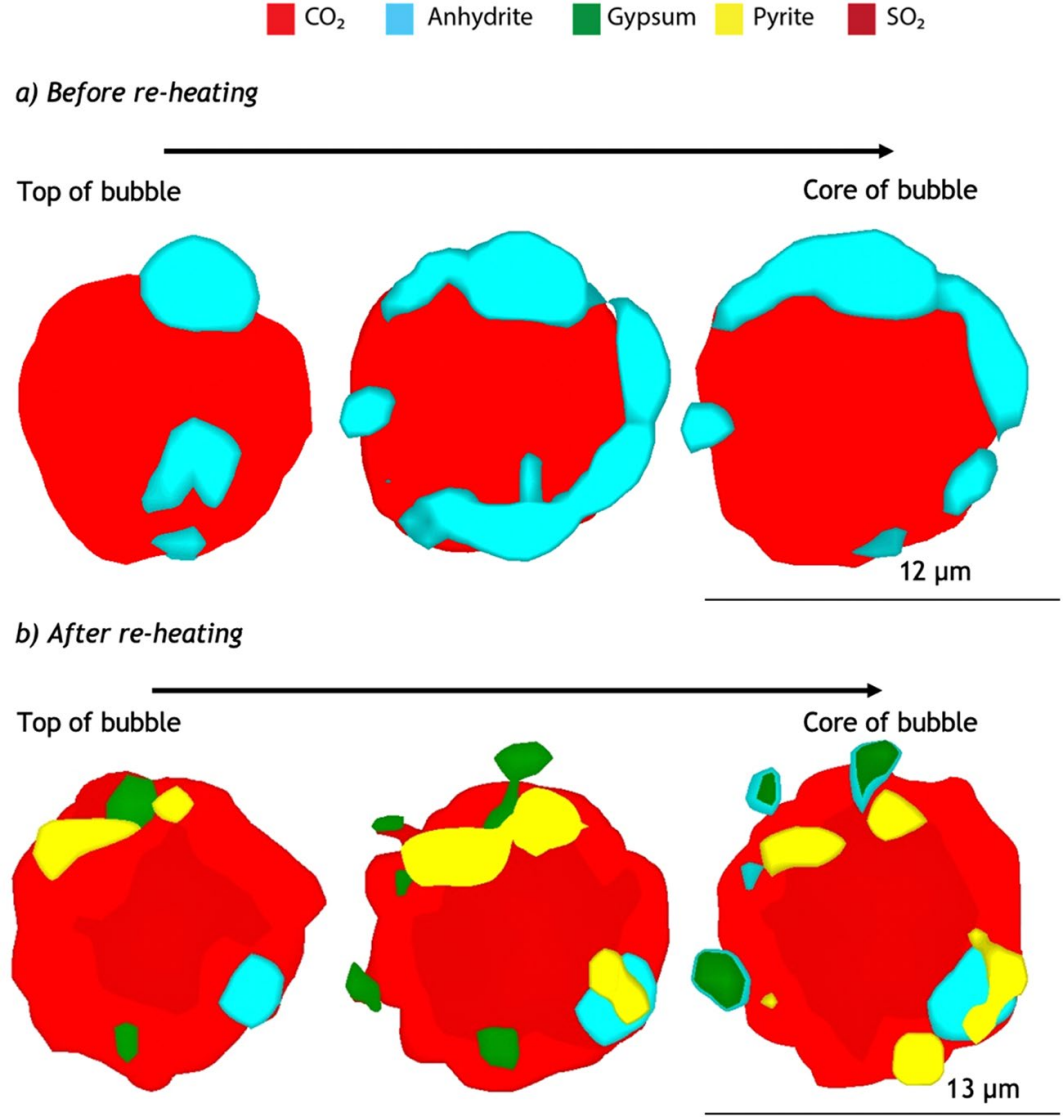
Horizontal sections through 3D scans of a vapour bubble (Brhm-33) (**a**) before and (**b**) after re-heating. Prior to re-heating, the bubble contains two main phases: CO_2_ gas (red) in the centre of the bubble surrounded by a discontinuous shell of anhydrite (CaSO_4_, light blue). Following re-heating, part of the anhydrite hydrates to gypsum (green). More S enters the bubble and forms pyrite (yellow) and SO_2_ gas. Although the SO_2_ peak is detected only in the central part of the bubble (dark red), SO_2_ is considered to be fully mixed with CO_2_ in the entire volume not occupied by the mineral phases; in fact, in the light red region, the intensity of the SO_2_ peak becomes as low as the spectral noise intensity, so only CO_2_ peaks can be detected. Water is not detected in the bubbles except in the molecular form attached to gypsum. For a given mineral, color shadows are due to variations in Raman signal intensity and indicate that part of the signal comes from slightly below, or above, the shown planar sections. All solid phases are connected to the bubble walls. In few shown sections (e.g. in the top portion of the bubble), some crystals appear to float in the gas phase but, in reality, are connected to the wall (upper or lateral) of the bubble. Due to the lack of evidence supporting a brine phase, the elements in the bubbles migrate from the glass, with no secondary or external sources. This also implies that the H_2_O, CO_2_, S, and Fe in the vapour bubble represents a “loss” from the glass and are major components of the total volatile budget. Three-D scans and the horizontal sections were generated using Renishaw’s WiRE software.

In every bubble from Mount Cayley and Garibaldi Lake, CO_2_ is the main gas phase (Fig. 2a). Estimating between 630 and 1400 ppm CO_2_ in the glass before re-heating (1.3 × 10^−11^ to 2.0 × 10^−10^ g; Supplementary Table S2), and assuming that the whole bubble is filled with fluid CO_2_, Raman acquisitions indicate that between 44 and 88% of the total CO_2_ is found in the vapour bubble, before re-heating (Supplementary Table S2). Negligible water contents were found within the bubbles of three samples. Anhydrite (CaSO_4_) is the dominant solid phase present in every bubble (Fig. 2a). Three-D scans prior to re-heating show that the bubble is filled with CO_2_, while anhydrite forms a partial shell with discrete crystals^4,13^ (Fig. 3a).

Volumetrically, CO_2_ and anhydrite occupy 88% and 12% of the bubble, respectively (Table 1). Using 3D scan volumes, glass contents of 1150 ppm S (Table 1), and 10.7 wt% CaO^21^, 58% of the total (glass + bubble) CO_2_, 20% of the total S and 0.5% of the total CaO is found in the vapour bubble (Table 1).

**Table 1:** Volumetric and elemental proportions calculated from 3D scans and ImageJ.

		**Re-heated MIs**		**Naturally glassy MIs**		
		**Brhm 33 before**	**Bhrm 33 after**	**MMA100**	**MMA200**	**MMA300**
**Bubble radius**	**cm**	0.0006	0.00065	0.0009	0.0014	0.0013
**Volume of bubble**	**cm**^**3**^	6.30 × 10^-10^	8.46 × 10^-10^	3.67 × 10^-9^	1.15 × 10^-8^	9.70 × 10^-9^
**Cumulative volume of phases inside bubble**	**cm**^**3**^	**9.03 × 10**^**-10**^	**1.15 × 10**^**-9**^	**3.05 × 10**^**-9**^	**1.41 × 10**^**-8**^	**9.50 × 10**^**-9**^
**Density**						
CO_2_ (Fermi doublet)	g/cm^3^	0.34	0.29	0.12	0.23	0.23
CaSO_4_	g/cm^3^	2.97	2.97	2.97	2.97	2.97
CaSO_4_ × 2H_2_O	g/cm^3^		2.3		2.3	2.3
FeS_2_	g/cm^3^		5	5	5	5
CaCO_3_	g/cm^3^			2.71	2.71	2.71
SO_2_	g/cm^3^		0.42			
**Volumetric proportions**						
CO_2_	vol%	87.7	83.5	80.2	88.2	76.1
CaSO_4_	vol%	12.3	2.8	2.1	0.9	0.7
CaSO_4_ × 2H_2_O	vol%		5.5		0.9	1.5
FeS_2_	vol%		6.9	9	1	18.5
CaCO_3_^b^	vol%			8.6	9	3.2
SO_2_	vol%		1.3			
CO_2_	cm^3^	7.92 × 10^-10^	9.60 × 10^-10^	2.45 × 10^-9^	1.24 × 10^-8^	7.24 × 10^-9^
CaSO_4_	cm^3^	1.11 × 10^-10^	3.22 × 10^-11^	6.41 × 10^-11^	1.27 × 10^-10^	6.65 × 10^-11^
CaSO_4_ × 2H_2_O	cm^3^		6.33 × 10^-11^		1.27 × 10^-10^	1.43 × 10^-10^
FeS_2_	cm^3^		7.94 × 10^-11^	2.75 × 10^-10^	1.41 × 10^-10^	1.76 × 10^-9^
CaCO_3_	cm^3^			2.62 × 10^-10^	1.27 × 10^-9^	3.04 × 10^-10^
SO_2_	cm^3^		1.50 × 10^-11^			
	sum	**9.03 × 10**^**-10**^	**1.15 × 10**^**-9**^	**3.05 × 10**^**-9**^	**1.41 × 10**^**-8**^	**9.50 × 10**^**-9**^
**Mass proportions**						
**CO**_**2**_	g	**2.69 × 10**^**-10**^	**2.78 × 10**^**-10**^	**2.94 × 10**^**-10**^	**2.86 × 10**^**-9**^	**1.66 × 10**^**-9**^
**CaSO**_**4**_	g	**3.30 × 10**^**-10**^	**9.56 × 10**^**-11**^	**1.90 × 10**^**-10**^	**3.77 × 10**^**-10**^	**1.98 × 10**^**-10**^
CaO	g	1.36 × 10^-10^	3.94 × 10^-11^	7.84 × 10^-11^	1.55 × 10^-10^	8.14 × 10^-11^
SO_3_	g	1.94 × 10^-10^	5.62 × 10^-11^	1.12 × 10^-10^	2.22 × 10^-10^	1.16 × 10^-10^
S	g	7.77 × 10^-11^	2.25 × 10^-11^	4.48 × 10^-11^	8.88 × 10^-11^	4.65 × 10^-11^
	sum	3.30 × 10^-10^	9.56 × 10^-11^	1.90 × 10^-10^	3.77 × 10^-10^	1.98 × 10^-10^
**CaSO**_**4**_ **× 2H**_**2**_**O**	g		**1.45 × 10**^**-10**^		**2.92 × 10**^**-10**^	**3.28 × 10**^**-10**^
CaO	g		4.74 × 10^-11^		9.51 × 10^-11^	1.07 × 10^-10^
SO_3_	g		6.76 × 10^-11^		1.36 × 10^-10^	1.52 × 10^-10^
S	g		2.71 × 10^-11^		5.44 × 10^-11^	6.10 × 10^-11^
H_2_O	g		3.04 × 10^-11^		6.11 × 10^-11^	6.86 × 10^-11^
	sum		1.45 × 10^-10^		2.92 × 10^-10^	3.28 × 10^-10^
**FeS**_**2**_	g		**3.97 × 10**^**-10**^	**1.37 × 10**^**-9**^	**7.05 × 10**^**-10**^	**8.79 × 10**^**-9**^
Fe	g		1.85 × 10^-10^	6.39 × 10^-10^	3.28 × 10^-10^	4.09 × 10^-9^
S	g		2.12 × 10^-10^	7.34 × 10^-10^	3.77 × 10^-10^	4.70 × 10^-9^
	sum		3.97 × 10^-10^	1.37 × 10^-9^	7.05 × 10^-10^	8.79 × 10^-9^
**CaCO**_**3**_	g			**7.11 × 10**^**-10**^	**3.44 × 10**^**-9**^	**8.24 × 10**^**-10**^
CaO	g			3.98 × 10^-10^	1.93 × 10^-9^	4.62 × 10^-10^
CO_2_	g			3.13 × 10^-10^	1.51 × 10^-9^	3.62 × 10^-10^
	sum			7.11 × 10^-10^	3.44 × 10^-9^	8.24 × 10^-10^
**SO**_**2**_	g		**6.28 × 10**^**-12**^			
S	g		3.19 × 10^-12^			
O	g		3.09 × 10^-12^			
	sum		6.28 × 10^-12^			
**Weight percent**						
Bubble mass	g	5.99 × 10^-10^	9.23 × 10^-10^	2.57 × 10^-9^	7.67 × 10^-9^	1.18 × 10^-8^
CO_2_	wt%	45	30.18	23.61	56.98	17.16
CaO	wt%	23	9.41	18.57	28.37	5.51
Fe	wt%		20.02	24.89	4.28	34.67
H_2_O	wt%		3.30		0.80	0.58
S (total)	wt%	13	28.71	30.32	6.78	40.72
**Glass composition**		Brhm 33 before ^a^	Bhrm 33 after	MMA100	MMA200	MMA300
CO_2_	ppm	695	600	1200	1328	1372
S	ppm	1154	420	2640	2857	2538
CaO	wt%	10.71	10.71	9.2	9.3	9.12
Fe	wt%	4.16	4.16	6.98	5.93	6.68
H_2_O	wt%	0.8	0.8	2.57	2.89	2.73
**Melt inclusion parameters**						
MI volume	cm^3^	1.07 × 10^-7^	1.07 × 10^-7^	5.54 × 10^-8^	1.15 × 10^-6^	1.51 × 10^-7^
MI volume w/o bubble	cm^3^	1.06 × 10^-7^	1.06 × 10^-7^	5.23 × 10^-8^	1.14 × 10^-6^	1.41 × 10^-7^
glass density	g/cm^3^	2.6	2.6	2.6	2.6	2.6
mass of glass w/o bubble ^c^	g	2.75 × 10^-7^	2.75 × 10^-7^	1.36 × 10^-7^	2.95 × 10^-6^	3.67 × 10^-7^
CO_2_ in glass	g	1.91 × 10^-10^	1.65 × 10^-10^	1.63 × 10^-10^	3.92 × 10^-9^	5.04 × 10^-10^
S in glass	g	3.18 × 10^-10^	1.15 × 10^-10^	3.59 × 10^-10^	8.44 × 10^-9^	9.32 × 10^-10^
CaO in glass	g	2.95 × 10^-8^	2.94 × 10^-8^	1.25 × 10^-8^	2.75 × 10^-7^	3.35 × 10^-8^
Fe in glass	g	1.15 × 10^-8^	1.14 × 10^-8^	9.50 × 10^-9^	1.75 × 10^-7^	2.45 × 10^-8^
H_2_O in glass	g	2.20 × 10^-9^	2.20 × 10^-9^	3.50 × 10^-9^	8.54 × 10^-8^	1.00 × 10^-8^
**Glass + bubble phases**						
total CO_2_	g	4.61 × 10^-10^	4.43 × 10^-10^	7.69 × 10^-10^	8.29 × 10^-9^	2.53 × 10^-9^
total S	g	3.95 × 10^-10^	3.80 × 10^-10^	1.14 × 10^-9^	8.96 × 10^-9^	5.74 × 10^-9^
total CaO	g	2.96 × 10^-8^	2.95 × 10^-8^	1.30 × 10^-8^	2.77 × 10^-7^	3.41 × 10^-8^
total Fe	g	1.15 × 10^-8^	1.16 × 10^-8^	1.01 × 10^-8^	1.75 × 10^-7^	2.86 × 10^-8^
total H_2_O	g	2.20 × 10^-9^	2.23 × 10^-9^	3.50 × 10^-9^	8.54 × 10^-8^	1.01 × 10^-8^
**Total MI composition (glass + bubble)**						
CO_2_	ppm	1698	1614	5654	2809	6893
S	ppm	1436	1384	8360	3033	15623
CaO	wt%	10.76	10.74	9.55	9.37	9.30
Fe	wt%	4.16	4.23	7.45	5.94	7.80
H_2_O	wt%	0.80	0.81	2.57	2.89	2.75
**Percent of phase in bubble, relative to MI total**						
CO_2_	% in bubble	58	63	79	53	80
S	% in bubble	20	70	68	5.8	84
CaO	% in bubble	0.46	0.29	3.44	0.79	1.90
Fe	% in bubble		1.59	6.30	0.19	14
H_2_O	% in bubble		1.37		0.07	0.68

MI refers to melt inclusion. Volume of bubble refers to the volume calculated using the radius measured under the microscope, and assuming a spherical bubble. However, to avoid overestimation, the cumulative volumes of the solid and gas phases in the vapour bubble are considered to be more representative of the bubble volume. Volumetric proportions refer to the total space occupied by each specific phase within the bubble. Mass proportions are calculated using the density of each phase. The density of SO_2_ was calculated using the ideal gas law. The quantity of each element or oxide was added to the glass total (proportions within the glass)^21^ to determine the total composition of the MI, including the bubble components. For re-heated bubbles, there is a systematic increase in all elements and oxides following re-heating, providing concrete proof that elements diffuse from the glass into the bubble during re-heating. For the glassy Mount Meager MIs, the bubbles contain a significant amount of major and volatile elements, suggesting that elemental diffusion is a naturally occurring process in glassy MIs.

^a^ The concentration of CO_2_ and S in the glass of Brhm-33 before re-heating was calculated using the mass of CO_2_ and S that entered the vapour bubble following re-heating (Supplementary Table S2).

^b^ The peaks of Mg-bearing carbonate were often detected, but the amount of Mg were below the detection limit. For simplicity, we assume all carbonates are CaCO_3_.

^c^ The mass of bubble was subtracted from the mass of the glass to calculate the mass of CO_2_, CaO, S, FeO, and H_2_O in the glass.

Each MI was re-heated at the same rate and temperature steps to avoid water loss from the glass^24^. The bubble persisted throughout the experiment and, upon quench, the diameter increased by up to 30% (volume increase up to 120%). The extent of bubble size increase is dependent on the formation of solid phases, and the total amount of CO_2_ in the glass that is able to diffuse above the closure temperature^4,10^. In all samples, the density of CO_2_ in the bubble decreased by up to 50% after re-heating, while the mass of CO_2_ increased, suggesting CO_2_ diffusion from the glass to the bubble during re-heating (Table 1). If the bubble contained only fluid CO_2_, 67 to 90% of the total CO_2_ would be sequestered within the bubble following re-heating (Supplementary Table S2). These are considered maximum values due to the presence of solid phases.

As a result of re-heating, anhydrite hydrates to gypsum (CaSO_4_·2H_2_O), resulting in a volume increase^25^ (Table 1). Additional compositional changes following re-heating include the introduction of new S-bearing species. In most samples, sulphur diffuses from the melt and forms gaseous SO_2_ in the bubble, which mixes with CO_2_ (Figs. 2b, 3b). Native S and Fe-sulphides (pyrite and polymorphic marcasite) appear in several bubbles following re-heating. Due to the similar Raman spectra between pyrite and non-magnetic pyrrhotite (Fe_1-x_S), we cannot exclude the possibility of pyrrhotite within the bubbles^26^ (Fig. 2b). The new S-bearing species have contrasting oxidation states, suggesting an intermediate oxidizing-reducing environment after reheating, which is consistent with previous findings of rapid *f*O_2_ re-equilibration during MI re-heating^19^. Nonetheless, these S-bearing phases suggest saturation with respect to sulphide and sulphate, and subsequent degassing from the glass to the bubble.

Three-D scans reveal the physical re-distribution of elements and phases within the bubble after re-heating: CO_2_ and SO_2_ gas are mixed throughout the bubble, while crystals of gypsum, anhydrite and pyrite generally nucleate on the bubble rim and grow unevenly in size and orientation (Fig. 3b). Mount Cayley and Garibaldi Lake MIs contain low water contents, between 0.1 and 1 wt%, with one inclusion containing 2 wt%^21^. Therefore, the persistence of anhydrite following re-heating is either due to its partial breakdown, and/or availability of H_2_O in the melt/glass as it is a limiting factor in the formation of gypsum. Volumetrically, the re-heated bubble contains 84% CO_2_, 1% SO_2_, 7% pyrite, 6% gypsum, and 3% anhydrite (Table 1). Of the MI total (glass + bubble), the amount of S in the bubble increases to 70%, while pyrite and gypsum sequester 1.6% FeO and 1.4% H_2_O, respectively (Table 1). The amount of CO_2_ in the bubble, relative to the total MI, increases to 63%. The mass of CO_2_ gained by the bubble is balanced by the mass of CO_2_ lost by the glass (Supplementary Table S2). Using 3D scans, and assuming mass conservation (amount of S gained by the bubble is balanced by the amount lost by the glass), the initial S content of the glass is approximately 1150 ppm, and decreases to 420 ppm following re-heating due to diffusion to the bubble (Table 1); this corresponds to a 64% loss of S from the glass. Prior to re-heating, the calculated S content of the glass suggests near saturation conditions, but this becomes masked by the low S concentration following re-heating.

### Glassy MIs

Mount Meager MIs contain 1200-1370 ppm CO_2,_ 2500-2800 ppm S, 9.1-9.3 wt% CaO, 5.9-6.9 wt% FeO and 2.6-2.9 wt% H_2_O in the glass^21^ (Supplementary Table S3). The amount of CO_2_ in the vapour bubbles, assuming that fluid CO_2_ is the only phase present, represents 45 to 87% of the total MI content (2400-8250 ppm; Supplementary Table S2).

**Figure 4:**
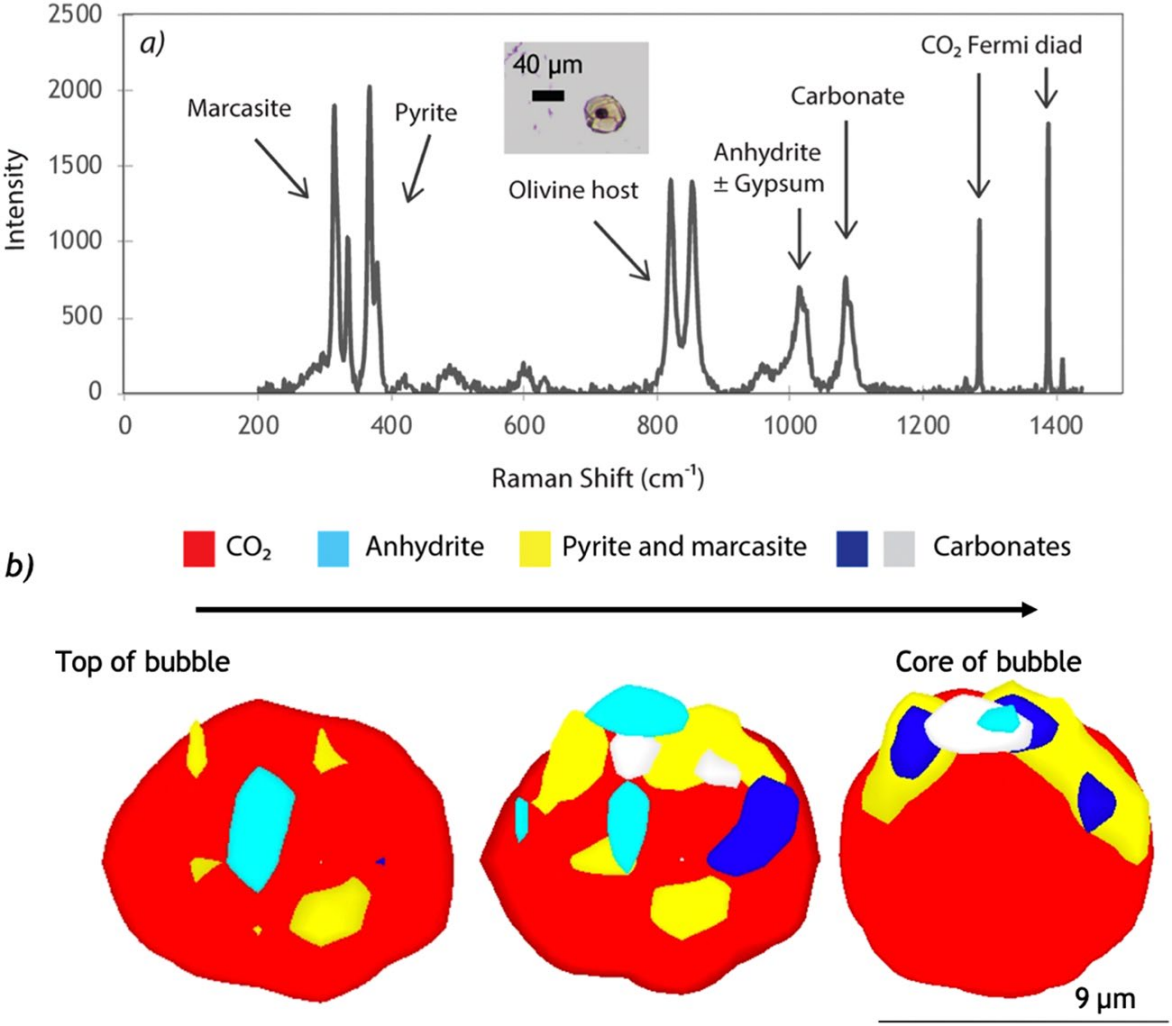
Raman spectrum and horizontal sections through a 3D scan of the same vapour bubble hosted in a glassy MI (MMA100) from Mount Meager. (**a**) Raman spectrum reveals that CO_2_ is accompanied by pyrrhotite or pyrite (with polymorphs of marcasite), anhydrite, gypsum, and carbonate. Inset photo corresponds to the analysed vapour bubble. (**b**) Horizontal sections through a 3D scan of the same vapour bubble (MMA100). Pyrite and marcasite (yellow), anhydrite (light blue), and Mg-Ca-Fe carbonates (dark blue and white/grey) form a partial shell around the bubble, with some crystals elongating toward the center of the bubble. The presence of Fe sulphides in MMA100 is proof of Fe migration from the glass during natural conditions. As in Fig. 3, water is not detected in the bubbles except in the molecular form attached to gypsum. All solid phases are connected to the bubble walls. Due to the lack of evidence supporting a brine phase, the elements in the bubbles migrate from the glass, with no secondary or external sources. This also implies that the H_2_O, CO_2_, S, and Fe in the vapour bubble represent a “loss” from the glass and are major components of the total volatile budget. Three-D scans and the horizontal sections were generated using Renishaw’s WiRE software.

Raman spectra of bubbles hosted in glassy MIs reveal significant proportions of both C- and S-bearing solids (Fig. 4a). Every bubble contains carbonates: Mg-calcite ((Mg,Ca)CO_3_)) is the dominant phase, while siderite (FeCO_3_) is less abundant. The acquired Raman spectra show relatively large carbonate peaks because of the presence of (Ca-, Mg-, and Fe-bearing) solid solutions. Therefore, in estimating the volumes of the different phases contained in the bubbles, we assumed for simplicity that the carbonates are primarily CaCO_3_. Anhydrite and pyrite (and/or non-magnetic pyrrhotite) are always present. Some samples also contain marcasite and/or nahcolite (NaHCO_3_). Samples MMA200 and MMA300 contain both anhydrite and gypsum.

Three-D scans show a partial shell of either pyrite/marcasite or carbonate with a centre filled with CO_2_ gas (Fig. 4b; Table 1). Volumetrically, CO_2_ occupies between 76 and 88% of the bubble. Anhydrite and gypsum are volumetrically minor, with a combined proportion below 3 vol% (Table 1). The partial pyrite shell in MMA 100 (Fig. 4b) and MMA300 occupies 9 and 19 vol% of the bubbles, respectively, whereas the bubble of MMA200 has a partial carbonate shell occupying 9 vol%. The bubble represents up to 3.4% CaO, up to 84% S and 6 to 14% FeO of the total MI (Table 1). Including carbonates, the total amount of CO_2_ in the MI rises to 2800-6900 ppm, with 53-80% in the bubble.

## Discussion

### Magmatic conditions beneath the GVB

Using the major oxide, volatile and trace element contents of Mount Cayley, Garibaldi Lake and Mount Meager MIs corrected for post-entrapment crystallisation^21^, the magmatic conditions beneath each centre can be calculated (Supplementary Table S3). Using olivine-melt equilibria^27^, entrapment temperatures range from 1090 to 1150 °C for Mount Cayley and 1120 to 1180 °C for Garibaldi Lake; these values are consistent with re-heating experiments, specifically the temperature when the last daughter crystal dissolved (1000 to 1150 °C for all MIs). Entrapment temperatures range from 1100 to 1140 °C for Mount Meager MIs. The pressure of MI entrapment and/or host crystallisation was calculated using a volatile saturation model^28^. Using only the glass composition of CO_2_ and H_2_O, pressure values range between 100 and 170 MPa for Mount Cayley, 130 and 310 MPa for Garibaldi Lake, and 140 and 200 MPa for Mount Meager^21^. The pressure values, assuming that the whole bubble is filled with gaseous CO_2_ (Fermi diad in Raman spectra), increase to 210-370 MPa for Mount Cayley, 140-580 MPa for Garibaldi Lake and 160-620 MPa for Mount Meager. Finally, after taking into account the volume of solids and gaseous phases (measured by 3D scans), the pressure values increase further. The entrapment pressure for an individual MI following re-heating increases from 140 to 225 MPa. Pressure values for two glassy MIs increase from 610 to 665 MPa, and from 210 to 375 MPa.

**Figure 5:**
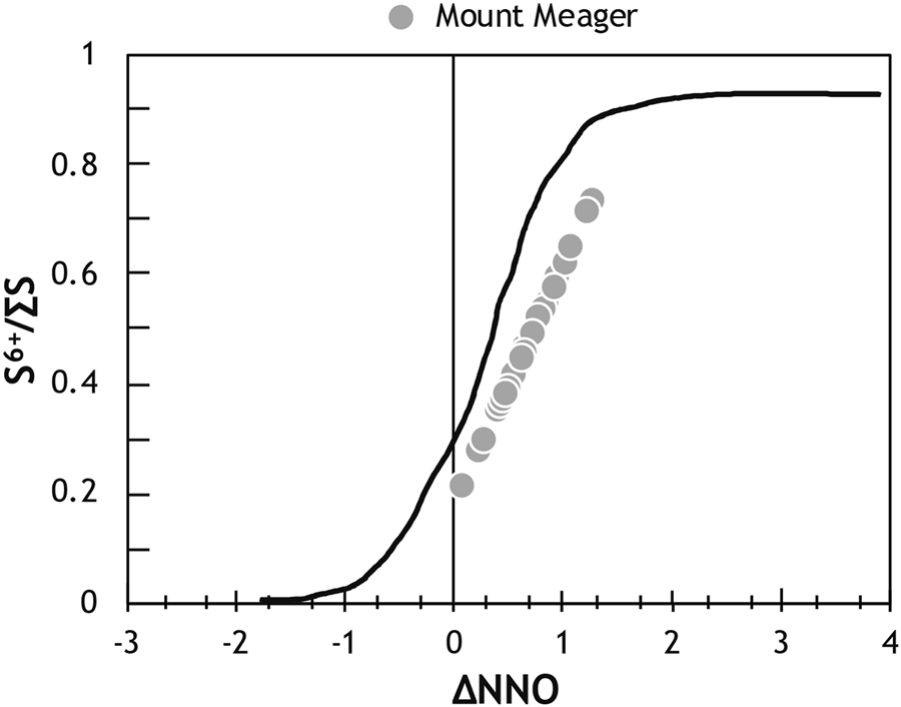
The speciation of sulphur within the glassy portion of Mount Meager MIs^21^. Represented by the relative proportion of S^6+^ and the sum of S^6+^ and S^2−^, S^6+^/∑S is calculated from the S peak^29^ and shows that the glass contains roughly equal proportions of S^2−^ and S^6+^. The corresponding *f*O_2_ was calculated from S^6+^/∑S^29,63^. Both the S^6+^/∑S and the *f*O_2_ suggest an intermediate oxygen fugacity, and a system saturated with respect to sulphide (pyrite; S^2−^) and sulphate (anhydrite; S^6+^). However, after considering the amount of S sequestered by the vapour bubble, we expect the S^6+^/∑S and *f*O_2_ to be higher, and in favour of sulphate saturation. The black curve corresponds to the relationship between S^6+^/∑S and relative oxygen fugacity (∆NNO) determined using basaltic MIs^63^.

The relationship between S speciation in the glass and *f*O_2_ is only applicable to Mount Meager MIs since re-heating experiments rapidly reset the oxidation state of MIs^19,29,30^. Using only the glass content of S, the ratio of S^6+^/∑S varies between 0.2 and 0.8, suggesting the co-existence of sulphide and sulphate species, and corresponds to *f*O_2_ between NNO and NNO + 1.5 (Fig. 5). However, due to the presence of S-rich solids in the bubble, the proportion of sulphate is likely much higher. Finally, the Fe^3+^/∑Fe, which is an additional proxy for the oxidation state of magma, was calculated using the glass composition, temperature and *f*O_2_, and ranges between 0.15 and 0.26^31^ (Supplementary Table S3). Such elevated ratios suggest an oxidised magma; due to re-equilibration between the melt and the bubble through the diffusion of Fe, these values should also be considered as minimum estimates.

### Does re-heating modify the composition of a melt inclusion?

Re-heating experiments reveal new phases that form through equilibration reactions between the melt and the bubble (gas) phase. The observation of these phases helps to understand the petrology of bubbles hosted in naturally glassy MIs (Reactions 1-5; Table 2).

**Table 2:** Chemical reactions within the vapour bubble.

Reaction	
1	FeS (*sulfide,melt*) + CaO (*melt*) + 2 O_2_ (*melt*) → CaSO_4_ (bubble) + FeO (*melt*)
2	CaSO_4_ (*bubble*) + 2H_2_O (*melt*) → CaSO_4_∙2H_2_O (*bubble*)
3	2FeS (*melt*) + Fe_2_O_3_ (*melt*) → FeS_2_ (*bubble*) + 3FeO (*melt*)
4	CaSO_4_ (*bubble*) → CaO (*bubble*) + SO_3_ (*bubble*)
5	CaSO_4_ (*bubble*) + CO_2_ (*melt or bubble*) → CaCO_3_ (*bubble*) + SO_3_ (*bubble*)

Brackets on the reactants side indicate the source of the chemical species (either the glass or the bubble) while brackets on the product side indicate the destination. Reaction 1 describes the precipitation of anhydrite within MI vapour bubbles, and the associated reduction of the glass. Reactions 2-4 explain the formation of gypsum, pyrite, SO_2_ gas within vapour bubbles following MI re-heating. With the exception of Reaction 4, all reactions listed here can be extrapolated to natural conditions to explain the similar solid phase assemblage in the vapour bubbles of glassy MIs. Regardless of crystallised or glassy MIs, the formation of phases within the vapour bubble is associated with significant changes in the glass, including major and volatile elemental loss as well as a lower Fe^3+^/ΣFe and *f*O_2_.

Decompression and cooling of the magma leads to crystallisation and volatile saturation (first boiling). The stability field of anhydrite also expands to lower *f*O_2_ as pressure decreases due to oxygen consumption from the melt/glass^32–35^ (Reaction 1, Table 2). Following MI entrapment, decompression promotes CO_2_ exsolution and flooding of the vapour bubble, as well as anhydrite precipitation within the bubble (Reaction 1, Table 2). The natural hydration of anhydrite to gypsum occurs slowly at low temperatures (60 to 100 °C), but is accelerated at elevated temperatures^25^. The only source of H_2_O is from the melt/glass, since no water was observed in the bubble before re-heating (Reaction 2; Table 2). Previous observations of H_2_O loss from MIs during experimental re-heating have shown that H^+^ rapidly diffuses from the MI through point defects in the olivine host, contemporaneously with *f*O_2_ equilibration with the external environment^19,20,26^. Our experiments, conducted at atmospheric pressure, show that the melt can also lose H_2_O to the bubble (where it is stored as gypsum) over timescales of <10 minutes when heated to 1200 °C, which are the experimental conditions. Extrapolation to natural magmatic conditions suggests that H_2_O diffusion from the melt to the vapour bubble can occur over geologically short timescales (e.g., during magma decompression).

The highly reducing conditions within the heating apparatus (*f*O_2_ < 10^−10^ atm) cause Fe diffusion and re-equilibration from the glass to the bubble, a process termed here as “Fe-migration”, which can be rapid enough to occur over timescales of <10 minutes during re-heating (Reaction 3, Table 2). The migration of Fe from the glass to the bubble is revealed by the formation of Fe-sulphides. Pyrite and marcasite are metastable polymorphs at temperatures <450 °C^36^. Above this temperature, only pyrite is stable. Three-D scans show the presence of well-formed cubic pyrite, implying rapid growth during re-heating (Fig. 3b). Marcasite is only present in a few vapour bubbles and always associated with pyrite; it is often represented by broad peaks in the Raman spectra, suggesting poorly formed crystals. Mobilisation of Fe^3+^ leads to a significant change in *f*O_2_. Consistent with previous findings of rapid (≤20 hours) *f*O_2_ re-equilibration during re-heating^19^, Fe_2_O_3_ in the glass reduces to FeO (Reaction 3, Table 2). Coupled with the formation of anhydrite in the bubble (Reaction 2, Table 2), these reactions lead to a higher proportion of Fe^2+^ in the glass, thereby lowering the Fe^3+^/ΣFe. In such MIs, the Fe content of the glass and the calculated *f*O_2_ is not representative of the magma at depth.

### Total volatile budget

Re-heating experiments were designed to reduce water loss^19,20,24^, leading to negligible proportions of H_2_O in re-heated bubbles. Minimal water was also detected within vapour bubbles of naturally glassy MIs (Table 1).

Focusing on MIs with 3D scans, and taking the carbonate crystals into account, the total amount of CO_2_ in glassy Mount Meager MIs ranges from 2800 to 6900 ppm. Without considering the carbonates, the total MI CO_2_ is 2400 to 6700 ppm. Both estimates suggest an original MI internal pressure of ≥0.5 GPa^28^ (CO_2_-vapour saturation pressure) in line with anhydrite stability^32^. This is significantly higher than pressure estimates based on the glass CO_2_ content alone^21^ (max 0.4 GPa), highlighting the importance of the volatile content of the bubble. Moreover, the slightly alkalic nature of Mount Meager basaltic magmas^21^ supports a higher CO_2_ solubility^37^, and a magmatic origin for the total CO_2_.

The presence of anhydrite in Mount Cayley and Garibaldi Lake bubbles before re-heating, and in Mount Meager bubbles, implies sulphate saturation within the melt following bubble nucleation. The first observations of igneous anhydrite were from the 1982 El Chichon and the 1991 Pinatubo eruptions^38,39^. Experimental investigations have shown that sulphates are the primary and stable S species at *f*O_2_ > NNO^29,39–41^. At 1 atm, anhydrite is stable until 1200 °C at NNO -0.8 to +0.2^41–43^, while anhydrite-saturated melts contain up to 2300 ppm S at 200 MPa, NNO and above 1000 °C^41^. Previous studies of primitive olivine-hosted MIs from subduction zones have reported co-genetic anhydrite inclusions, further supporting sulphate saturation conditions of arc basalts^44^. The aforementioned magmatic conditions calculated from Mount Meager MIs are likely underestimated due to the sequestration of S and Fe in the vapour bubble. Adding the sulphur from the bubble yields a total S content between 0.3 and 1.6 wt% (Table 1). Previous experiments have shown that up to 0.5 wt% S can be dissolved in oxidised basaltic melts at 500 MPa^45^. After considering the reduction of the melt due to Fe-migration, we estimate that the actual *f*O_2_ of the MI would be much higher, and consistent with a basaltic melt saturated with sulphate^46^. Therefore, stable anhydrite within the bubble suggests bubble nucleation between 1000 and 1200 °C^32–34^, which is consistent with the calculated temperatures.

The presence of Fe-sulphides in the Mount Meager bubbles suggests Fe-migration is a naturally occurring process that is likely accelerated during re-heating experiments. Pyrite in vapour bubbles has been previously reported but as a minor constituent, and as a product of bubble-brine interaction resulting in a concentration of solid phases only at the bubble-glass rim^13,16^. Similarly, dehydration experiments on olivine-hosted MIs have shown that H^+^ loss promotes the re-distribution of sulphur from the glass to the fluid or vapour bubble^44,47^. Both hypotheses imply that sulphur within vapour bubbles is a secondary process. As a whole, the results of our experiments and the absence of liquid water in the bubble rule out pyrite precipitation under hydrothermal conditions. Therefore, we show that the diffusion of S from the melt, and the formation of S-bearing phases in the bubble, represents the necessary exsolution processes as the glass achieves saturation with respect to sulphate. Similar reactions have been proposed to explain the high amount of SO_2_ gas released during the climatic 1991 Pinatubo eruption, where the reaction between SO_2_ and H_2_O in a magma produces sulphides, supercritical H_2_S fluid, and anhydrite^48^. In a magma, sulphide saturation creates sulphur globules, and sulphate saturation results in anhydrite crystallisation or SO_2_ gas. Our results show that significant sulphur exsolution can also occur in the MI leading to the precipitation of S-bearing phases within the bubble.

### The excess sulphur problem

Since the 1991 Pinatubo eruption, there has been considerable attention paid to excess degassing by volcanoes^49–52^. Scaling the difference in sulphur between MIs (pre-degassed melt) and groundmasses (degassed melt) to the volume of erupted magma provides an estimation of the mass of sulphur released during an eruption. Comparisons of such “petrologic” estimates with remote sensing measurements of SO_2_ emissions have shown that the concentration of pre-eruptive sulphur in MIs are commonly too low to account for the total mass of SO_2_ released during an eruption^46,50,53^. Excess degassing has been attributed to degassed, but non-erupted, magma, the presence of a deep sulphur-rich immiscible fluid^46,51,52,54^ and/or an underestimation of the sulphur content measured in MIs^52,53^. Indeed, petrological estimates using bubble-bearing MIs have only considered the sulphur dissolved in the melt/glass. The present study shows that, when using bubble-bearing MIs, the sulphur trapped within the vapour bubble reasonably accounts for the excess sulphur emitted during an eruption. The application of our findings to cases of excess degassing assumes that sulphur saturation was achieved, and exsolution promoted the formation of solids within the bubble. Examples of previously calculated excess degassing scenarios determined using bubble-bearing MIs include the explosive 1986 basaltic eruption of Chikurachki (Russia), which released 0.7 Mt of SO_2_, while the petrological estimate of SO_2_ was only 0.5 Mt^52,54^. Assuming that the vapour bubble of Chikurachki MIs contained similar quantities of sulphur to those measured here, the total amount of sulphur would increase by up to 84%, thereby matching the amount of SO_2_ released during the eruption. No excess degassing would therefore be required. Conversely, the 1991 eruption of Pinatubo emitted 20 Mt of SO_2_ into the atmosphere, while the petrological estimate of SO_2_ was only 0.28 Mt^38,52,55^. With 84% of sulphur in the bubble, petrological estimates only increase to 0.96 Mt. However, this eruption was dacitic and our results pertain to basaltic magma. This suggests that felsic MIs may sequester more sulphur within the vapour bubbles, which is consistent with experimental observations that show the sulphur content at sulphide saturation (SCSS) correlates positively with the glass FeO content^56^. Furthermore, as felsic magmas cool, they exsolve metal-rich aqueous fluids that record the first stage in the evolution of hydrothermal fluids and ore deposits^57^. As such, vapour bubbles hosted within felsic MIs are also likely to sequester chalcophile metals that partition from the late-stage melt, and precipitate within the bubble as sub-micron scale S-rich minerals.

### Implications

The evolution of magma from its source, ascent through the mantle and crust, and eruption at the surface is inevitably associated with dramatic changes in its chemistry. Olivine-hosted MIs, although record the magma composition and storage parameters at the time of entrapment, are subject to post-entrapment modifications, such as the nucleation of vapour bubbles and daughter crystals, that result in discordance with the original magma. Regardless of whether the MI is re-heated or naturally glassy, our study highlights the significance of the vapour bubble, and its sequestration of major and volatile elements that originate from the melt/glass.

Re-heating reverses daughter crystal nucleation and yields the original MI composition. Using Raman spectroscopy and 3D scans, we show that significant compositional changes occur within the MI during re-heating. Over timescales of <10 minutes and temperatures between 400 and 1200 °C, the melt/glass can lose up to 65% CO_2_ and up to 64% S to the bubble. The resulting glass composition is unrepresentative of the original magma, and yields incorrect calculations of *f*O_2_, pressure and volatile saturation. Bubbles hosted in naturally glassy MIs contain 45 to 87% of the total CO_2_ content in the bubble. Relative to the MI total, up to 84% S, 3.4% CaO, and 14% Fe is found in the bubble. This shows that elemental diffusion is a natural process in a MI and can operate during magmatic decompression.

The calculated magmatic conditions for GVB magmas change significantly when considering the bubble composition. Using the mass of CO_2_ in the bubble, the pressure values for both re-heated and glassy MIs increased by 80 to 130%. Using 3D scans and adding the solid phase composition to the MI total, pressure values further increase by up to 80%. Following the reduction reactions in Table 2, we would expect the S and Fe in the bubble to be added back to the glass as their oxidised forms, thereby increasing the overall Fe^3+^/ΣFe and *f*O_2_ of the MI to be correlative with sulphate-saturation conditions. By accounting for the bubble, GVB magmas are revealed to be oxidised and sulphate saturated.

The diffusion of S from the glass poses significant implications for excess degassing: accounting for sulphur in the bubble could explain the discrepancy between the petrological method and remote sensing measurements for excess sulphur estimates based on basaltic bubble-bearing MIs.

In the case of crystallised MIs, high pressure homogenisation has the potential to yield MIs that are representative of the magma at depth. Whether the MI is crystallised, re-heated or glassy, the composition of the vapour bubble should be analysed to obtain an accurate composition of the magma at MI entrapment. Overall, our study highlights the importance of the vapour bubble in MI studies as it is a hidden reservoir for major and volatile elements that contribute to the total budget, and cautions the use of re-heated MIs to be representative of the original magma.

## Methods

### Reheating experiments

Individual olivine crystals from Mount Cayley and Garibaldi Lake were reheated at the Laboratoire Magmas et Volcans (LMV) in Clermont-Ferrand, France. Olivines were double polished, mounted on sapphire discs, and progressively, and rapidly, heated to a constant temperature, between 1150 and 1200 °C using a Vernadsky-type heating stage containing a 1 atm gas-tight sealed furnace cooled by water. Pure He gas, purified by Zr metal at 700 °C, was circulated through the furnace to maintain reducing conditions (fO_2_ < 10^−10^ atm) and prevent olivine oxidation. Temperatures inside the furnace were recorded by a type-S thermocouple welded to the sample holder. Samples were held at a given temperature for 1 minute and, as such, each experiment was approximately 10 minutes in total. Short duration experiments optimally minimise water loss due to diffusion^20,24^ and limit the decrease in volatile solubility that occurs due to the pressure decrease within softened crystals at high temperature^58^. The maximum temperature of the re-heating apparatus was 1200 °C and, in every case, the bubble remained, meaning a fully homogenised MI was not possible. Many apparatuses have similar maximum temperatures; therefore, the bubble likely remains in many re-heating situations. Nonetheless, the aim of the study was to investigate the composition of the vapour bubble in equilibrium with the silicate melt and, as such, the sample was rapidly quenched once the bubble began to move inside the inclusion, indicating a molten silicate melt.

### Raman spectroscopy

The composition of all vapour bubbles was analysed using Raman spectroscopy (LMV, France). In the case of Mount Cayley and Garibaldi Lake, the bubble was analysed before and after reheating in order to track any compositional changes. Spectra were collected using an InVia confocal Raman micro-spectrometer manufactured by Renishaw and equipped with a 532 nm diode laser (200 mW output power), a Peltier-cooled CCD detector of 1040 ×256 pixels, a motorised XYZ stage and a Leica DM 2500 M optical microscope. Scattered light was collected via a back-scattered geometry. Laser power was periodically checked and reduced to 8 mW on the sample surface; thus, the power was lower than this value within the bubble. A grating of 2400 grooves mm^−1^, a 100x microscope objective and a 20-μm slit aperture (high confocality setting) were used, which resulted in spectral resolution better than 0.4 cm^−1^ and in lateral and vertical spatial resolutions of approximately 1 and 2-3 μm, respectively, near the sample surface. Vertical resolution decreases with depth mainly due to light refraction at the air/glass/bubble interfaces. Daily calibration of the wavelength was performed based on the 520.5 cm^−1^ peak of Si. The spectra were recorded using the WIRE 4.2 software in the wavenumber range 60-1410 cm^−1^, which includes the vibrational frequencies characteristic for mineral phases, such as carbonates, sulphates, sulphides and silicates, for CO_2_ and SO_2_ gases^59^, and for the alumino-silicate network domain of glasses. Spectra were also collected in the 2800-3900 cm^−1^ region to detect H_2_O and/or OH molecules. Presence of thin films of liquid water inside the bubble was difficult to detect in some bubbles, especially in the smallest ones (<10 µm), because of fluorescence or contamination of the spectrum by more intense glass water bands. Nonetheless, negligible water contents were measured. Acquisition time for a single analysis ranged between 60 and 120 seconds.

Before (3D) volume acquisitions, we performed depth profiles to define the vertical dimension of the 3D map. The selected volume was generally within 30 µm depth below the sample surface, so the signal/noise ratio remained high. The step size between acquisition points was 1-1.5 µm on the x- and y-axis and 1-2 µm on the z-axis. The total number of acquisitions for a single 3D map varied from 7000 to 16700. The acquisition time was set to 20 s/point and the spectra were centered at 850 cm^−1^. For spectra treatment, we first identified and removed cosmic rays, then we carefully chose the most appropriate polynomial baseline correction and applied it to the entire dataset. Then, the main peaks that best represent the different phases were selected to build the 3D map. Due to the small size and transparency of the bubbles, the collected spectra generally show a mixture of signals coming from different phases. Therefore, for 3D reconstruction, the variation of the relative intensities of peaks belonging to different phases must be carefully evaluated to correctly assign Raman acquisitions to distinct phases. When the errors associated with 3D data acquisition, spectra treatment and subsequent calculations are considered, the estimated total error on the obtained volatile budget is <30%. This error does not include possible overestimation of the volume of solid phases that is expected if the size of crystals is lower than the spatial resolution of the instrument (i.e., 1-2 μm).

### Calculating the CO_2_ density

The main gas phase is CO_2_ with two strong peaks at ~1284 and 1387 cm^−1^; this is referred to as the Fermi doublet, or diad^59,60^. The difference between the two main peaks (Δ) is used to calculate the density of CO_2_^61^. The mass of CO_2_ is found by multiplying the density by the volume of the bubble measured under the microscope. The mass fraction of CO_2_ in the bubble relative to the glass can be calculated knowing the mass inside the bubble, the glass CO_2_ concentration, the volume of the bubble and the volume of the MI (Supplementary Table S2). The amount of CO_2_ estimated using this approach, and assuming that CO_2_ is the only phase present inside the bubble, can be compared to the total amount of CO_2_ obtained from the volumetric analysis of the bubble, which allows quantification of both gaseous CO_2_ and carbonates.

### Quantitative volumetric analysis

Renishaw’s WiRE software was first used to process the acquired 3D data and create 2D slices of the bubble volume. Then, volumetric analyses were performed, and volumes were measured using ImageJ software^62^. The 2D slices, or cross sections, were taken at equal distances depending on the size of the bubble (every 1 or 1.5 µm). Volumes between slices (called spherical segments) were calculated using the distance between each slice and the radius of each slice. For the top and bottom segment of the bubble (spherical cap), the volume was calculated using the equation for a hemisphere. Summing up the total volume of all spherical segments, plus the 2 spherical caps, yielded values close to volume estimates assuming a spherical bubble. In most cases, the former method yielded lower values than the latter and is considered to more accurately describe the exact bubble shape and volume. From here, a global scale was applied to each set of slices, and the area occupied by each phase was calculated. Finally, the percentage by volume (vol%) of a phase (mineral or gaseous) in the bubble was calculated knowing the relative area percent of that phase in each slice, multiplying it by the relative vol% of the associated spherical segment, and summing the vol% of that phase in each segment.

From the mass of the solid and gaseous phases, obtained by multiplying their volumes by appropriate density values, the total amount of CO_2_, H_2_O, S, Ca, and Fe were calculated (knowing the weight % of the element/molecule in a mineral). For an element that is present in different phases (e.g., S in sulphides, sulphates and SO_2_, or C in CO_2_ and carbonates), summing up the mass of that element in all phases yielded the total mass of the element in the bubble (Table 1).

Details concerning Electron Microprobe and SIMS analyses can be found as Supplementary Material. The full corrected MI dataset including S, H_2_O and CO_2_ contents can be found in Supplementary Table S3.

## Supplementary information

Supplementary information.

Supplementary information.
