# Supplementary material for: Melt inclusion vapour bubbles: the hidden reservoir for major and volatile elements
Source: Sci Rep. 2020 Jun 3;10:9034. doi: 10.1038/s41598-020-65226-3 (PMC7270139; doi:10.1038/s41598-020-65226-3)
Supplement: Supplementary file 1 — Supplementary information. [file 41598_2020_65226_MOESM1_ESM.pdf]

Manuscript title: Melt inclusion vapour bubbles: the hidden reservoir for major and volatile elements.

Authors: Swetha Venugopal*, Federica Schiavi, Severine Moune, Nathalie Bolfan-Casanova, Timothy Druitt and Glyn Williams-Jones

Supplementary methods

Additional information concerning samples

We chose primitive (Fo > 80) basaltic olivine-hosted melt inclusions (MIs) that have been thoroughly investigated for their major, volatile and trace element contents and magmatic conditions from 3 volcanoes along the Garibaldi Volcanic Belt in western Canada. The size and volume of bubbles and melt inclusions were estimated using a microscope under transmitted light and Leica imaging software. Melt inclusion volumes were assumed to be ellipsoidal and the two observable axes were measured. The best estimate for the third unobservable axis was approximated using the smaller ellipsoidal axis measured in the microscope. Bubble volumes were assumed to be spherical and calculated using measured diameters that were accurate to 2 µm. The associated errors for size and measurements are less than 10 %. We obtain similar bubble volumes when using the arithmetic mean of the two horizontal axes (e.g. Tucker et al., 2019).

Eight crystallised MIs from Mount Cayley and Garibaldi Lake were 15 – 30 µm, contained a vapour bubble that occupied < 10 vol % of the inclusion and daughter crystals that did not intersect the bubble (Supplementary Figure S1). Seven MIs from Mount Meager were large (20 – 40 µm), glassy and commonly contained a shrinkage bubble occupying less than 10 % by volume (Supplementary Figure S2b). Samples with vapour bubbles that occupied > 10 vol % were avoided as they likely do not represent primary vapour bubbles or were entrapped as a separate phase, i.e, pre-existing bubbles (Moore et al., 2015). For ease of analysis, all MIs were chosen based on their size, orientation of daughter minerals and clarity of the vapour bubble. Smaller MIs were chosen for more efficient Raman spectra acquisition but also to avoid chemical gradients in the melt which is commonly observed for larger MIs (Lu et al., 1995).

This study will be supplemented with data from Venugopal et al. (2020) who used the same melt inclusion samples from Mount Cayley, Garibaldi Lake and Mount Meager. They analysed a set of MIs for major, volatile and trace elements using Electron Microprobe and LA ICP-MS at LMV. Water and CO_2 concentrations in the glass were analysed using the Secondary Ion Mass Spectrometer (SIMS) at CRPG Nancy. Raman analyses determined the amount of CO_2 in the bubble. Detailed methodology of analytical instrumentation is provided below.

Electron Microprobe

Major and volatile (S and Cl) elemental compositions of melt inclusions, host crystals, and matrix glasses were analyzed at LMV using a SX-100 CAMECA electron microprobe with a 15 kV accelerating voltage. Mineral analyses were performed using a 15 nA focused beam that was defocused to 10 or 20 µm during glass analyses to reduce Na loss. In order to collect the most precise data and reduce volatile loss during analysis, the beam was blanked regularly with a Faraday cup and 5 measurements were taken at 20 s intervals. Volatile analyses were measured with a 40 nA sample current and a 50 s acquisition time using the LPET diffraction crystal for S and Cl. Sulphur speciation (S^{6+}/S_{total}) was obtained from the S Kα peak shifts relative to the peak shift of barite and sphalerite, temperature and linear regression coefficients (Wallace and Carmichael, 1994) in order to estimate the oxygen fugacity of the melt inclusions following the method of Jugo et al. (2005). Speciation was measured at least 3 times in Mount Meager melt inclusions, as they are the only primary, non-re-heated inclusions in this study. This method cannot be applied to re-heated inclusions since the internal oxygen fugacity is reset to that of the re-heating stage during experiments. The precisions of the electron microprobe analyses (2σ) are better than 5 % for major elements, except for MnO, Na_2O and K_2O, which had an EMP precision < 10 %. The approximate 2σ precision for S and Cl is 4 % and 7 %, respectively. The full corrected dataset is found in Supplementary Tables S3.

Since sample mounts required carbon-coating for microprobe analyses, SIMS analyses were preformed first to avoid any C contamination.

Secondary Ion Mass Spectrometer (SIMS)

Water and CO_2 values of the melt inclusions were analyzed using the CAMECA IMS 270 Ion Probe (SIMS) at the Centre de Recherches Pétrographiques et Géochimiques (CRPG) in Nancy, France with a 15 µm beam size for all analyses. Indium-mounted samples were gold-coated and pre-sputtered with a 10 kV Cs + primary beam of 10 to 15 nA. No sample mounts experienced any carbon contamination before SIMS (i.e., carbon coating or diamond polish). Background C counts were monitored to ensure stable signals during analyses. Before and after each analytical session, a series of well characterised basaltic glass standards (KL2G, Etna, Mount St Helens, M34, M35, M40, M43 and M48 (Bindeman et al., 2012; Kendrick et l., 2012; Jochum et al., 2006; Newman et al., 1988; Hauri et al., 2002; Kamenetsky et al., 2000 and Kamenetsky and Maas, 2002) were used for calibration of water and carbon contents (total range: 4.4 – 3172 ppm CO_2 and 0.015 – 5.7 wt% H_2O). Carbon dioxide and water contents of melt inclusions are found in Supplementary Table S3.

Supplementary figures and tables

Figure S1. Melt inclusions from this study. The olivine-hosted MI pictured in the top row is from Garibaldi Lake and was taken a) before and b) after re-heating experiments. The MI is 27 µm in diameter and contained a vapour bubble that was approximately 12 µm in diameter. Prior to re-heating, the MI contained daughter crystals as a result of post-entrapment modifications. The MI was re-heated using a Vernadsky-type heating stage until the crystals disappeared, the melt was molten and the bubble began to move. At this point, the sample was quenched. The bubble diameter increased slightly upon quench, to a diameter of 13 µm. Glassy Mount Meager MIs are shown in c) and d).

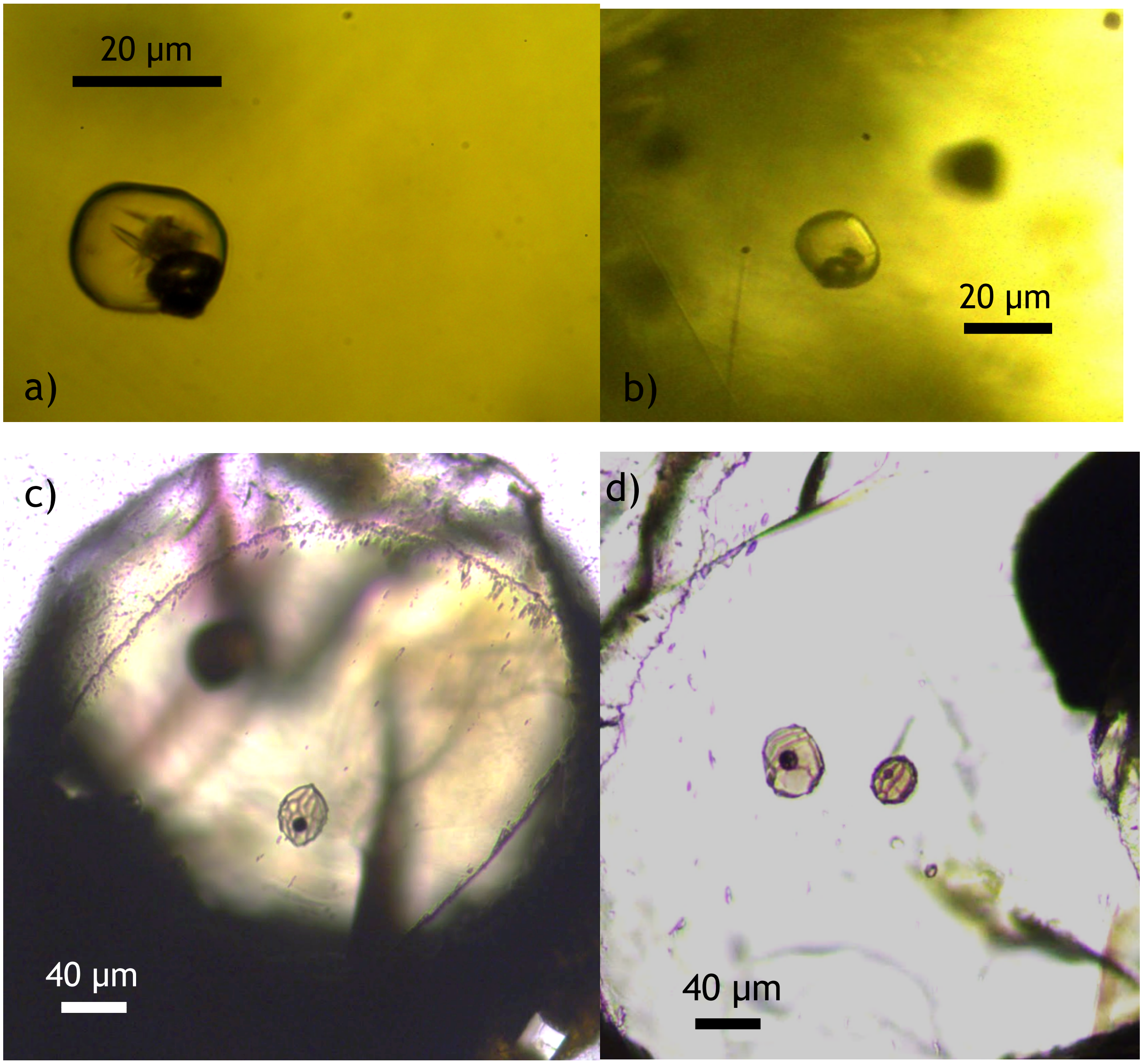

Table S1. Sample location and composition of basaltic whole rocks used in this study. VF denotes volcanic field. Most phenocrysts in the whole rock samples also occur as microlites in the groundmass. Olivine host size refers to the grain sized from which melt inclusion-bearing olivines were picked. Major oxides are listed in wt%, and whole rock compositions are renormalized to dry weight.

Refer to Excel sheet.

Table S2. Quantifying the amount of CO_2 in the vapour bubble using the Fermi diad. Volumes of vapour bubbles and melt inclusions given in cm^3. The size and volume of bubbles and melt inclusions were estimated using a microscope under transmitted light and Leica imaging software. Melt inclusion volumes were assumed to be ellipsoidal and the two observable axes were measured. The best estimate for the third unobservable axis was approximated using the smaller ellipsoidal axis measured in the microscope. Bubble volumes were assumed to be spherical and calculated using measured diameters that were accurate to 2 µm. In the case of vapour bubbles imaged in 3D (indicated by *), the cumulative volume of the phases within the bubble is taken as a more representative estimate of the bubble volume. The associated errors for these measurements (size and volume) is less than 10 %. Density of CO_2 was calculated using the Fermi diads and the equation from Wang et al. (2011). Mass of CO_2 in the bubble calculated assuming that fluid CO_2 is the only phase present in the bubble. For the re-heated samples, the difference between before and after re-heating are significant. The volume of the bubble and the contained mass of CO_2 increases while the density of CO_2 decreases. The calculated CO_2 total concentrations are considered to be maximum values. Inferred CO_2 in the glass before re-heating was calculated using the mass of CO_2 gained by the bubble as a result of re-heating. *indicates 3D scan was performed.

Refer to Excel sheet.

Table S3. Major oxide and volatile element composition (wt%) of the glassy portion of MIs in this study provided by Venugopal et al. (2020), and calculated magmatic conditions. Garibaldi Lake and Mount Cayley MIs were reheated to dissolve the daughter crystals back into the glass. Mount Meager MIs are naturally glassy. Reheating establishes equilibrium between MI and olivine host (KD = 0.30-0.33); Mount Meager MIs were corrected until the glass composition was in equilibrium with the host crystal. CO2 glassy refers to the glass content of CO_2. FeO*[a] and S ppm[b] refer to the MI total after adding the solid phase composition from 3D scans. Total CO_2 (glass + bubble)[c] is the sum of the CO_2 in the glass plus the mass detected within the bubble using Raman spectra. Total CO_2 (glass + bubble)[d] is the sum of the CO_2 in the glass plus the amount calculated using 3D scans. Fe ratios were calculated using the glass composition of Mount Meager MIs using the method by Kress and Carmichael (1991). Temperature, expressed in ˚C, was calculated using equation 13 from Putirka (2008). P (MPa)[e] refers to the pressure values calculated using the volatile saturation model by Papale et al. (2006) considering glass composition and the mass of CO_2 in the bubble. P (MPa)[f] refers to the pressure values calculated using the solid phase content in the bubble and the glass composition. Bold and asterisk (*) indicates a 3D scan of the bubble was performed.

Refer to Excel sheet.
